# Incidence, predictors, and clinical outcome of patients with undetected aneurysms on initial DSA after aneurysmal subarachnoid hemorrhage

**DOI:** 10.1016/j.bas.2026.107468

**Published:** 2026-10-07

**Authors:** Carolin Albrecht, Laura Aberle, Ann-Kathrin Joerger, Hraq Sarkis, Arthur Wagner, Tobias Boeckh-Behrens, Julian Schwarting, Christian Maegerlein, Jan Kirschke, Bernhard Meyer, Maria Wostrack

**Affiliations:** a Department of Neurosurgery, Technical University of Munich, School of Medicine, Klinikum Rechts der Isar, Germany; b Department of Neuroradiology, Technical University of Munich, School of Medicine, Klinikum Rechts der Isar, Germany; c Department of Neurosurgery, BG Unfallklinik Murnau, Murnau, Germany

## Abstract

**Introduction:**

Digital subtraction angiography (DSA) is the gold standard for diagnosing ruptured intracranial aneurysms in aneurysmal subarachnoid hemorrhage (aSAH). However, aneurysms may occasionally remain undetected on initial DSA, potentially delaying treatment and increasing the risk of rebleeding. The primary aim was the incidence of aneurysms missed on initial DSA; secondary aims were characteristics, diagnostic yield and outcome.

**Material and methods:**

We retrospectively analyzed patients with aSAH treated between March 2006 and October 2023 with initial negative DSA. Demographic, clinical and radiological characteristics were assessed. Neurological outcomes were evaluated using the Glasgow Outcome Scale and modified Rankin Scale and compared with a matched cohort of patients with immediate aneurysm detection.

**Results:**

Among 1221 patients with aSAH, 14 (1.1%) had initially negative DSA findings with subsequent aneurysm detection. Repeat DSA identified the aneurysm in eight patients (57.1%). MRI was performed in 13 patients and contributed to aneurysm detection in three cases (21.4%). A third DSA revealed aneurysms in two additional patients (14.3%), while one aneurysm was identified during surgical exploration. The mean interval between initial and repeat diagnostics was five days (range 1-14). Rebleeding occurred in three patients (21.4%) before aneurysm diagnosis. Delayed-detection aneurysms were significantly smaller than aneurysms identified on initial DSA (1.8 mm vs. 6.7 mm, p < 0.05).

**Discussion and conclusion:**

Ruptured aneurysms may be missed on initial DSA, particularly when small. Repeat DSA, complemented by MRI when appropriate, should be performed during the same hospital stay, even in perimesencephalic hemorrhage patterns, to avoid delayed treatment and adverse outcomes.

## Highlights

• Ruptured aneurysms are missed on initial DSA in 1.1% of aSAH cases.
• Missed aneurysms are significantly smaller than detected ones (1.8 mm, p < 0.05).
• Second Repeat DSA or MRI detects 78.5% of occult aneurysms within a mean of 10 days.
• Repeat DSA is recommended for all SAH patients with typical bleeding patterns.

## 1 Introduction

Aneurysmal subarachnoid hemorrhage (aSAH) is a lethal neurological disorder, with an estimated 500,000 cases occurring annually worldwide (Hughes et al., 2018). Despite a recent decline in global mortality rates, the impact on daily life of those affected remains substantial (Taufique et al., 2016) .

Clinical outcomes are primarily influenced by the initial Hunt and Hess (HH) score and early intervention to treat the ruptured aneurysm (Doukas et al., 2019). Delayed aneurysm occlusion bears the risk of early rebleeding with subsequent impairment of the HH grade and increasing blood clot volume leading to further sequela such as cerebral vasospasm, brain edema, infarction, and overall worse neurological outcomes. As a result, a study advocates for early aneurysm repair, ideally within the first 24 h, to minimize the risk of the complications and improve outcomes (Hostettler et al., 2023).

Delayed aneurysm treatment can occur for several reasons, including delays in hospital admission, transfers between healthcare facilities, or initial misdiagnosis (Neil-Dwyer and Lang, 1997). When SAH is confirmed by computed tomography (CT), it is critical to identify the underlying aneurysm to occlude the source of the bleeding. The gold standard for detecting a ruptured aneurysm is catheter-based digital subtraction angiography (DSA). However, in some cases, the initial DSA may fail to detect the aneurysm, posing a risk of an undetected and therefore untreated bleeding source.

Further diagnostic procedures, including magnetic resonance imaging (MRI) or even spinal MRI in selected cases, along with follow-up DSA, may eventually reveal the ruptured aneurysm. This diagnostic delay can be challenging because it increases the time to aneurysm occlusion, a factor known to influence clinical outcomes (Neil-Dwyer and Lang, 1997).

Considering this, the primary objective of this study was to determine the incidence of aSAH in which the ruptured aneurysm was not detected on the initial DSA in a large single-center cohort. Secondary objectives were to characterize the clinical and radiological features of these patients, including bleeding pattern and Fisher scores, HH grade, and aneurysm size, location and morphology, in order to identify factors potentially associated with non-detection, as well as to describe the diagnostic yield and timing of the follow-up examinations that ultimately led to aneurysm detection and to compare neurological outcome, measured by the Glasgow Outcome Scale (GOS) and modified Rankin Scale (mRS) at discharge and at follow-up, with a matched control group of patients whose aneurysm was identified on the initial DSA. In addition, we performed a structured review of the literature to better contextualize our findings.

## 2 Methods

### 2.1 Patient population

Patients treated for aSAH in our department between March 2006 and October 2023 were retrospectively analyzed to assess the timing of aneurysm detection. The advanced search function of the Picture Archiving and Communication System (PACS) was used to identify patients with initially negative DSA. In addition, the hospital database of aSAH cases since 2006 was reviewed. Patients with traumatic SAH, secondary causes, or perimesencephalic non-aneurysmal SAH were excluded from the analysis. Factors such as age, sex, medical history, Fisher CT grade, HH score, and bleeding pattern were evaluated as potential predictive factors. Aneurysm size, location, and configuration were also recorded.

For outcome analysis, Glasgow Outcome Scale and modified Rankin Scale scores were collected at both discharge and six-month follow-up. These data were extracted from the hospital's clinical documentation system.

For the control group, patients with comparable HH scores and Fisher grades at admission, along with immediate aneurysm detection, were chosen from the clinical database. The matching process considered factors such as sex, age (within a ±9-year range), treatment approach (clipping or coiling), and complications. Variations in treatment methods were permitted. The primary matching criteria were HH and Fisher grades.

At our institute, the diagnostic protocol for suspected SAH initially includes a CTA, followed by DSA. In cases where no vascular lesion is detected on initial DSA, magnetic resonance angiography (MRA) is performed within two to four days. If no aneurysm is identified, a second repeat DSA is performed after 10-14 days. However, in clinical practice, the timing and modality of follow-up imaging were ultimately determined on a case-by-case basis, considering individual patient factors and interdisciplinary decision-making. This variability may influence detection rates but also reflects in workflows actual clinical environments. This diagnostic pathway was applied in principle throughout the whole study period.

Over the study period, three successive generations of biplane angiography systems from the same manufacturer were in use. 3D rotational angiography was performed in most initial examinations. All DSA examinations were performed and evaluated by interventional neuroradiologists. However, given the long observation period of 17 years, angiographic and MR imaging technology evolved considerably and changes in institutional practice over time cannot be fully excluded.

### 2.2 Ethical approvement

This study received approval from the local Research Ethics Committee (186/20S). Since the analysis was retrospective and the data used could not be linked to specific individuals, the Ethics Committee determined that retrospective informed consent from the patients was not required. All procedures in the study were conducted in compliance with the relevant guidelines and regulations.

### 2.3 Statistical analysis

Statistical analysis was performed using SPSS software (Version 30.0.0.0). Continuous variables are presented as mean ± standard deviation (SD) or median and interquartile range (IQR), depending on the distribution of the data set. The statistical level of significance was set at 5% (p-value = 0.05).

Descriptive statistics were used to summarize patient demographics and clinical characteristics. Univariate analysis was performed to identify possible predictors of occult aneurysms. Additionally, a matched-pair analysis compared neurological outcomes between patients with occult aneurysms and those with immediate aneurysm detection. The Wilcoxon signed-rank test was used to evaluate differences in outcomes between the case and control groups.

### 2.4 Structured literature review

To identify studies on angiogram-negative aSAH, a comprehensive search on PubMed database was performed. The search strategy included the following keywords:

Subarachnoid Hemorrhage OR SAH, negative angiography OR initial negative angiography OR initial negative DSA OR undetected aneurysm OR occult aneurysm OR angiogram-negative.

The reference lists of selected studies were reviewed to identify additional relevant articles not captured in the initial search. We focused on peer-reviewed articles published in English or German between 2000 and 2024 that examined the incidence, etiology, and functional outcomes of angiographically negative SAH. Articles that were irrelevant to the topic or not available in full text were excluded. The included studies provided data on the incidence of occult aneurysms, the diagnostic efficacy of CTA or MRA and DSA, factors contributing for missed aneurysm detection, bleeding patterns, and, when available, functional outcomes of these patients.

## 3 Results

### 3.1 Retrospective analysis

#### 3.1.1 Patient characteristics

Between March 2006 and October 2023, a total of 1221 patients were treated for aSAH at our department. Of these, 14 (1.1%) patients initially had a negative angiography, and the ruptured aneurysm was revealed only in a subsequent follow-up diagnostic. For the control group, another 14 patients with aneurysm detection in the first angiogram and similar HH- and Fisher-grades were selected from our hospital database (see Table 1).

Of the 14 patients with initially negative angiography, ten were female (71.4%) and the median age at admission was 56 years (range 34-79 years). HH scores at admission ranged from 1 to 4, with 9 patients (64.3%) presenting with only mild symptoms (HH scores 1-2). The amount of bleeding on CT-scan was classified using the Fisher grading scale (Fisher et al., 1980): six patients (42.9%) were classified as Fisher 3, and another four patients (28.6%) were Fisher 4. Intraventricular hemorrhage was observed in 50% of the patients. The remaining four patients had either no blood (Fisher 1: 7%) or a Fisher grad 2 bleeding (21.4%).

**Table 1:** Summary of case group.

Patient no.	Age/sex	HH	Fisher	Size (diameter in mm)	Location	Mode of detection
1	49/F	2	4	1.8	ICA	2nd (DSA)
2	45/F	1	3	1.2	Acom	2nd (MRA)
3	54/F	1	3	<1 (blister)	P1	2nd (DSA)
4	58/F	4	3	1.3	Basilar artery	2nd (DSA)
5	64/M	2	3	1	Basilar artery	3rd (DSA)
6	79/M	3	4	4.5	Basilar artery	2nd (DSA)
7	71/F	3	4	<1 (blister)	Acom	2nd (DSA)
8	45/F	2	2	1.9	ICA	2nd (MRA)
9	61/F	4	4	2	M1	3rd (surgical exploration)
10	58/F	2	3	1	P1	2nd (DSA)
11	51/M	4	3	1.5	Acom	3rd (DSA)
12	63/M	2	2	1.6	ICA	2nd (DSA)
13	36/F	1	2	3	P1	3rd (DSA)
14	34/F	1	1	3	Pcom	2nd (MRA)

Acom, anterior communicating artery; ICA, internal carotid artery; M1, proximal middle cerebral artery; P1, proximal posterior cerebral artery; Pcom, posterior communicating artery.

At admission, 12 patients (85.7%) reported headaches, four patients (28.6%) exhibited signs of meningism, and five patients (35.7%) experienced nausea. Nine patients displayed a typical bleeding pattern with involvement of the basal cisterns, while in four cases (28.6%) blood was only detected in the perimesencephalic region. In one case (7%), SAH was detected by lumbar puncture and was not visible on initial CT scan. In our cohort, pre-existing medical conditions were identified as follows: migraine in two patients, hypertension in four patients, hypothyroidism in three patients, diffuse astrocytoma in one patient, coronary artery disease and atrial fibrillation (along with hypertension) in one patient, and liver cirrhosis (along with hypertension) in one patient.

Aneurysm size (measured as maximum diameter) ranged from 0.5 mm to 4.5 mm with a mean size of 1.8 mm ± 1.1 mm. Compared to the control group (mean aneurysm size: 6.7 mm), patients with undetected aneurysms on the initial angiogram had significantly smaller aneurysms (p < 0.05, *t*-test for independent samples). Aneurysms were in the anterior circulation in seven patients (50%) and in the posterior circulation in the remaining seven (50%). Aneurysms were predominantly localized in the basilar tip (three cases), the anterior communicating artery (three patients) and the internal carotid artery (three cases). Atypical aneurysm morphology was observed in nine patients (64.3%): four aneurysms (28.6%) were perforating artery aneurysms, comprising two basilar artery perforator aneurysms (BAPAs) and two P1 perforator aneurysms, four (28.6%) showed a blister-like morphology, and one (7.1%) was a dissecting aneurysm of the P3 segment. The remaining five aneurysms (35.7%) were saccular. Both BAPAs presented with a perimesencephalic bleeding pattern (see Table 2).

**Table 2:** Characteristics and comparisons of case group (no initial aneurysm detection) and control group (aneurysm detection in first angiogram).

		Case group (n = 14)	Control group (n = 14)	p
**Median age (years)**		56	52.5	
**Female Sex**		71.4%	71.4%	
**Hunt & Hess**	1	4 (28.6%)	4 (28.6%)	
	2	5 (35.7%)	5 (35.7%)	
	3	3 (21.4%)	3 (21.4%)	
	4	2 (14.3%)	2 (14.3%)	
	5	0 (0%)	0 (0%)	
**Fisher**	0	1 (7.1%)	1 (7.1%)	
	1	0 (0%)	0 (0%)	
	2	3 (21.4%)	3 (21.4%)	
	3	6 (42.9%)	6 (42.9%)	
	4	4 (28.6%)	4 (28.6%)	
**Mean Aneurysm size (diameter in mm)**		1.8 ± 1.1	6.7 ± 3	<0.001 (*t*-test two independent samples)
**Aneurysmsite**	*ICA*	3 (21.4%)	3 (21.4%)	0.418 (fisher exact test)
	*MCA*	1 (7.1%)	1 (7.1%)	
	*Acom*	3 (21.4%)	6 (42.9%)	
	*P1*	3 (21.4%)	0 (0%)	
	*Basilar art.*	3 (21.4%)	1 (7.1%)	
	*Pcom*	1 (7.1%)	2 (14.3%)	
	*PICA*	0 (0%)	1 (7.1%)	
**Mean time to treatment (hours)**		240 ± 262	7.9 ± 4.5	0.002 (*t*-test, two independent samples)
**Complications**	*Spasm*	8 (57.1%)	9 (64.3%)	1.0 (fisher exact test)
	*HCP*	3 (21.4%)	5 (35.7%)	0.678
	*Rebleed*	3 (21.4%)	0 (0%)	0.222 (fisher exact test)
**GOS at discharge**	*mean*	4.2	4.1	0.75
	**1**	1 (7.1%)	1 (7.1%)	
	**2-3**	1 (7.1%)	3 (21.4%)	
	**4-5**	12 (85.7%)	10 (71.4%)	
**mRS at discharge**	*mean*	1.7	1.9	0.587
	**0-3**	12 (85.7%)	11 (78.6%)	
	**4-5**	1 (7.1%)	2 (14.3%)	
	**6**	1 (7.1%)	1 (7.1%)	
**GOS follow-up**	*mean*	4.6	4.8	0.680
	**1**	1 (7.1%)	1 (9.1%)	
	**2-3**	1 (7.1%)	0 (0%)	
	**4-5**	10 (83.3%)	10 (90.1%)	
**mRS follow-up**	*mean*	1.3	0.9	0.670
	**0-3**	10 (83.3%)	10 (90.1%)	
	**4-5**	1 (7.1%)	0 (0%)	
	**6**	1 (7.1%)	1 (7.1%)	

Acom, anterior communicating artery; ICA, internal carotid artery; MCA, middle cerebral artery; P1, proximal posterior cerebral artery; Pcom, posterior communicating artery; PICA, posterior inferior cerebellar artery.

#### 3.1.2 Diagnostic work-up

Of the 14 patients, 13 (92.9%) with an initial negative DSA underwent MRI with MR angiography. MRI with TOF sequences revealed the aneurysm in three cases (21.4%). In eight other patients, a second DSA revealed the aneurysm (57.1%). Two patients underwent surgical inspection before undergoing a third DSA after previous unsuccessful diagnostic procedures. Of these, the aneurysm was found in one patient (7.1%). In the other case, the patient underwent a third DSA.

In total, two out of 14 patients had an aneurysm on a third DSA (14.3%), which eventually identified the aneurysm. Repeat angiography was pursued even in patients with a perimesencephalic bleeding pattern when the clinical presentation was severe, particularly in cases involving a high HH score, a large perimesencephalic blood burden or associated hydrocephalus.

The mean time interval between the initial and repeat angiogram was five days and ranged from one to fourteen days. The time from hospital admission to aneurysm detection varied between one and forty-two days, with a mean of ten days.

Of the 14 patients without aneurysm detection in the initial angiogram (case group), nine underwent surgical clipping (64.3%), whereas five were treated with endovascular coiling (35.7%). During hospitalization, ten patients (71.4%) experienced at least one complication, with vasospasm (57.1%), hydrocephalus (21.4%) and re-bleeding (21.4%) being the most common complications. Two patients required endovascular spasmolysis due to symptomatic vasospasm. Retrospectively, the aneurysm could be detected via MRI in three of the eight cases undergoing second DSA (37.5%, see (Table 1).

Seven out of the 14 cases occurred after the current-generation angiography system was introduced, including three of the four perforating artery aneurysms.

#### 3.1.3 Clinical outcome

At discharge, 12 patients (85.7%) had favorable outcomes measured with values according to GOS scales, GOS IV and V. Of them, 8 patients were completely symptom-free at time of discharge. The mean GOS at discharge was 4.2 ± 1.3 with a mean mRS-grade of 1.7 ± 1.9. One patient died during hospitalization, and another was discharged with GOS 2 and mRS 5. Eleven of the thirteen survivors returned to hospital for clinical follow-up (78.6%). The timing of the first follow-up examination varied across the group, ranging from two to nine months, with a mean duration of 4.5 months. At follow-up, the mean GOS score improved to 4.6 ± 0.9, and the mean mRS score was 1.3 ± 1.4.

In the control group, the mean GOS score at discharge was 4.1 ± 1.4 and the mean mRS at discharge was 1.9 ± 1,9. At follow-up examination, the control group had a mean GOS score of 4.8 ± 0.4 and mean mRS was 0.9 ± 1.4. Matched-pair comparisons revealed no significant differences in GOS scores (p = 0.75) or mRs (p = 0.68) at discharge between case and control group. Similarly, at the 6-month follow-up, no statistically significant discrepancies in GOS (p = 0.59) and mRs (p = 0.67) could be detected.

Although the outcomes were generally good despite the diagnostic delays, the findings should be interpreted as hypothesis-generating rather than conclusive due to the limited number of cases.

Fig. 1: Comparison of Glasgow Outcome Scale (GOS) and modified Rankin scale (mRS) scores between case and control group at discharge and 6-month follow-up (see Fig. 2).

**Fig. 1:**
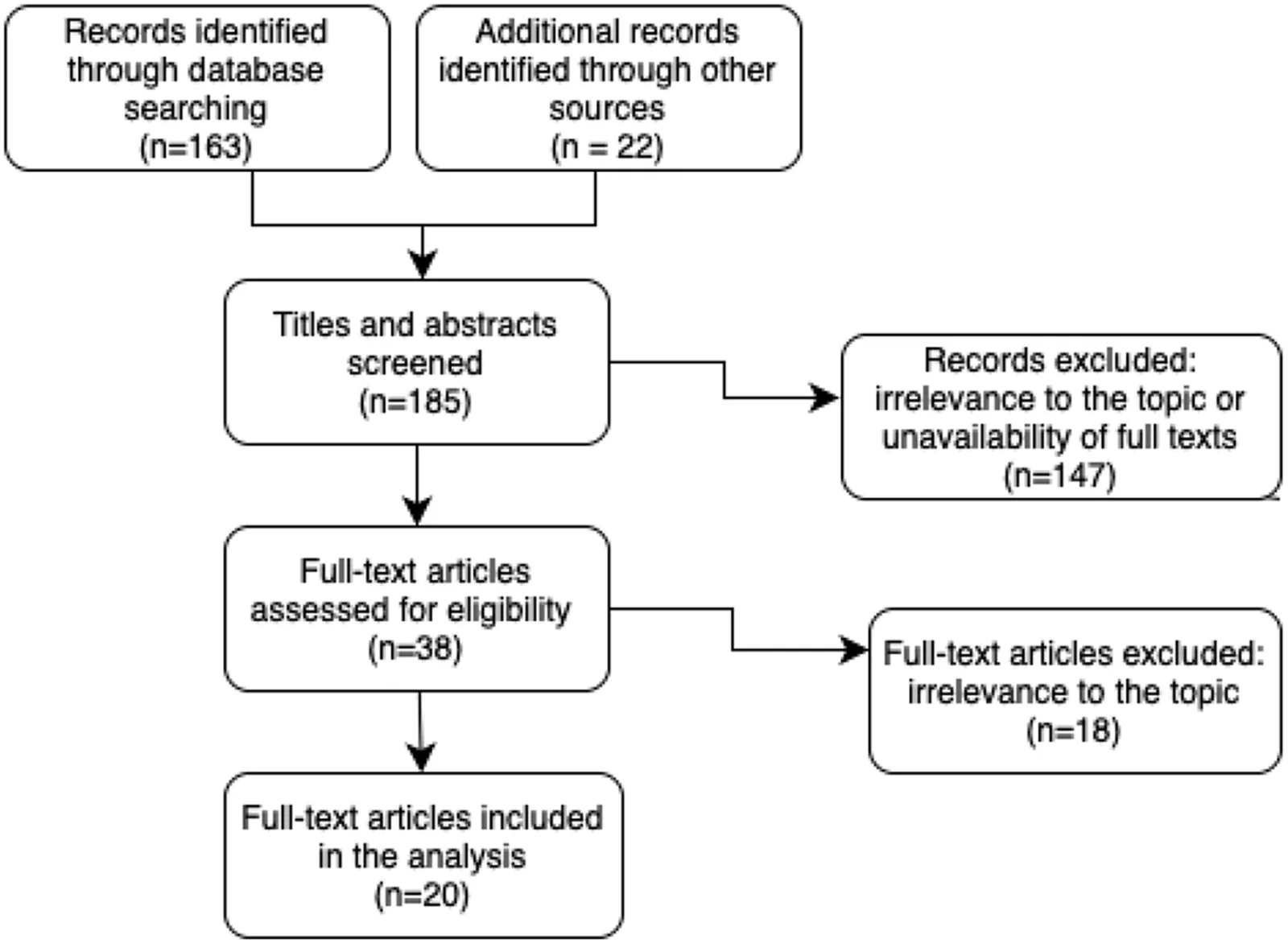
Flow chart: Study selection.

**Fig. 2:**
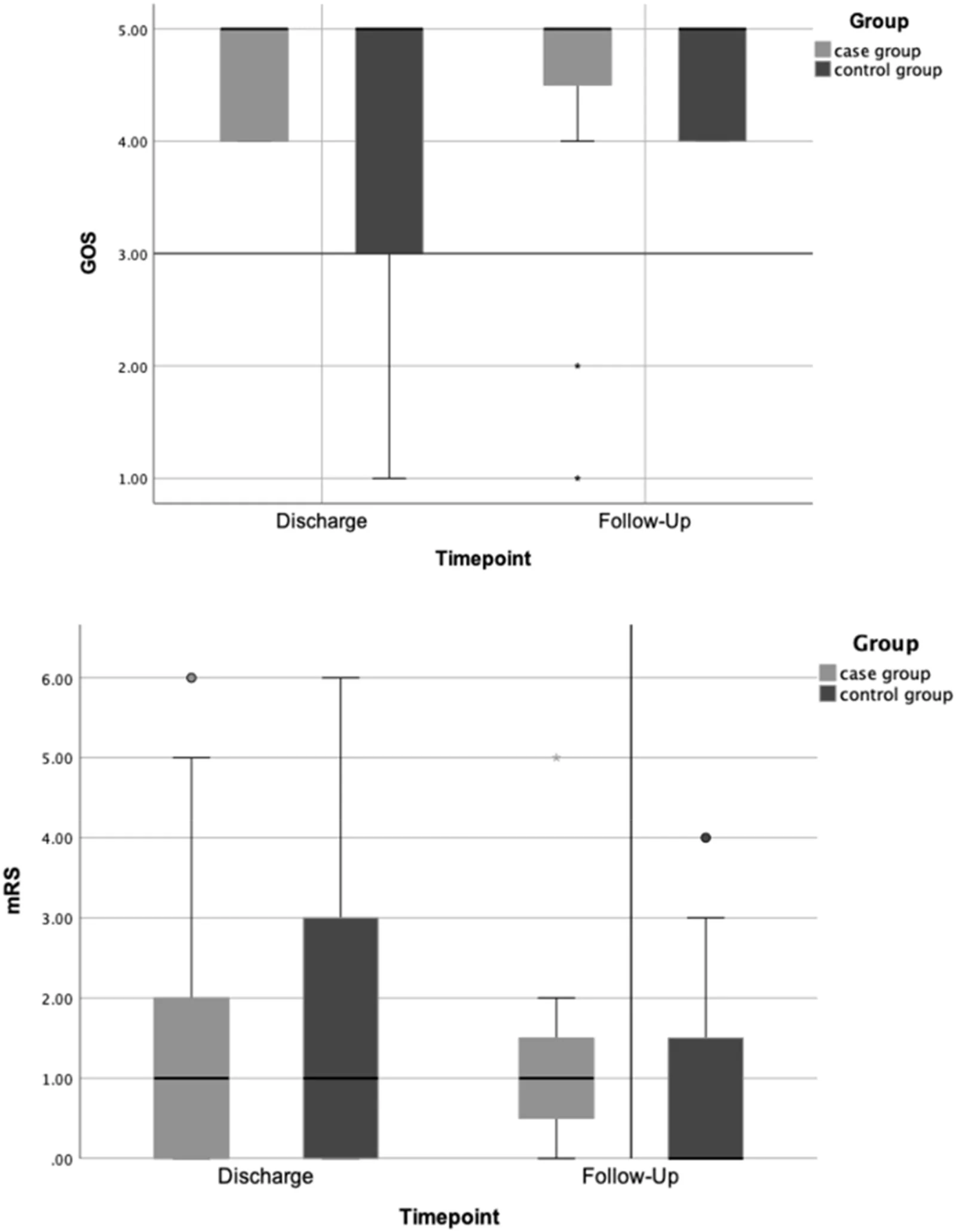
Comparison of GOS and mRS between the case and control group at discharge and 6-month follow-up.

### 3.2 Structured literature review

The initial search identified 163 articles, with an additional 22 articles identified through reference reviews. Of these, 38 were selected for full-text review, ultimately resulting in the inclusion of 20 studies in our qualitative analysis (see Table 3). The reported rates of misdiagnosis from the initial angiographic assessments ranged from 1.4% to 38%, with a mean rate of 7.8% ± 7.6%. To provide context to our findings, we calculated the incidence rate of aneurysms detected in follow-up examinations relative to the total cohort of SAH patients. Fourteen articles provided sufficient data from which we derived an overall incidence of 0.8% (range: 0.1-2.9%) for angiographically occult aneurysms. After excluding one case-report and one meta-analysis, a total of 139 patients without initial aneurysm detection were identified. Among these, 134 patients exhibited a typical SAH bleeding pattern, while only 5 patients (3.6%) presented with a perimesencephalic blood distribution. Most studies categorized patients into the following groups, based on the blood distribution on the initial CT scan: perimesencephalic (PM-SAH), characterized by blood restricted to the perimesencephalic or prepontine cisterns, and non-perimesencephalic or diffuse SAH (nPM-SAH), which involved diffuse hemorrhage throughout multiple cisterns, including the Sylvian and interhemispheric cisterns.

The initial vascular imaging modalities used in the studies were heterogenous: DSA (45%), DSA + MRA (15%), DSA + CTA (35%), CSA + MRA + CTA (5%). Procedural complication rates for DSA, reported by six studies, ranged from 0% to 5%, with a mean complication rate of 2% (Kumar et al., 2014; Moscovici et al., 2013; Ringelstein et al., 2013; Nesvick et al., 2022; Bashir et al., 2018; Bakker et al., 2014). Diagnostic follow-up was conducted primarily using DSA in nine articles, while four articles used both DSA and MRA, and six articles included CTA. The timing of the repeat imaging varied from four days to three months, although four studies did not report this information. A third DSA was performed in 10 studies, particularly in cases of vasospasm, rebleeding, or suspected aneurysmal bleeding pattern. One study (Nützel et al.) does not recommend a second repetition, while another (Nguyen et al.) supported the idea, considering it to be beneficial, especially for patients with diffuse bleeding patterns (Nützel et al., 2023; Nguyen et al., 2022).

In one study, repeat angiography after SAH revealed no aneurysms; however, MRI identified a potentially causative abnormality suspected to be a cavernoma (Khan et al., 2013).

Another study reported the detection of one aneurysm via conventional angiography, while a second was identified during surgical exploration, and spinal MRI revealed a cervical ependymoma (Little et al., 2007). A meta-analysis reported delayed diagnosis rates of 3.9% for DSA only, 1.7% for the combination of CTA and DSA, and 7.8% for DSA combined with MRI/MRA (Mohan et al., 2019). In one article, a second DSA revealed nine aneurysms, with an additional aneurysm identified during surgical exploration and another discovered by autopsy (Fontanella et al., 2011). Another study reported detection rates of 6.6% for repeat CTA, 11.7% for repeat DSA, and 10% for a group of patients who underwent both CTA and DSA (Wang et al., 2024). In the remaining studies included in our systematic review (75%), aneurysms were exclusively detected via repeat DSA.

Sixteen studies supported repeated DSA, although most recommended this approach primarily for patients with diffuse rather than perimesencephalic hemorrhage patterns. Moreover, two studies recommended non-invasive follow-up methods, such as CTA and MRA, in cases of perimesencephalic SAH. Notably, only five studies performed repeat angiographies for all patients with an initially negative angiogram (Moscovici et al., 2013; Ringelstein et al., 2013, 2015; Nützel et al., 2023; Inamasu et al., 2003).

The anterior communicating artery was identified as the most common site for aneurysms detected on repeat angiography in three studies (Nguyen et al., 2022; Inamasu et al., 2003; Jung et al., 2006).

Possible reasons for the failure to detect aneurysms on initial imaging included vasospasm, very small aneurysms, suboptimal imaging techniques, large blood volumes, arterial dissection, and thrombosis of the aneurysm (Moscovici et al., 2013; Ringelstein et al., 2013, 2015; Nguyen et al., 2022; Khan et al., 2013; Little et al., 2007; Fontanella et al., 2011; Inamasu et al., 2003; Jung et al., 2006; Chen, 2014; Xu et al., 2017). Functional outcomes were infrequently reported, with only one study providing specific data on neurological or functional outcomes in patients with angiogram-negative aSAH: among these, three patients showed favorable outcomes, two had mild to severe deficits and two died (Inamasu et al., 2003).

**Table 3:** Overview of included studies.

Author/year	Study type	Misdiagnosis rate	Incidence of occult aneurysms	Conclusion
**Kumar (2014)** (Kumar et al., 2014)	Prospective	5.1%	1.1%	Repeat angiogram in case of nPM-SAH
Yu (2012) (Yu et al., 2012)	Retrospective	7.1%	0.2%	Repeat angiogram in case of nPM-SAH (DSA for nPM-SAH, CTA for PM-SAH)
**Nützel (2023)** (Nützel et al., 2023)	Retrospective	3.9%		A second repeat angiogram is not recommended
**Moscovici (2013**) (Moscovici et al., 2013)	Retrospective	3.6%	0.7%	Repeat angiogram in case of nPM-SAH
**Ringelstein (2013)** (Ringelstein et al., 2013)	Retrospective	3.3%	0.1%	Repeat angiogram in case of PM-SAH
**Khan (2013)** (Khan et al., 2013)	Retrospective	2% (cavernoma)	0%	Not in favor of repeating angiogram (only in case of significant suspicion of vascular lesion)
**Inamasu (2003)** (Inamasu et al., 2003)	Retrospective	36%	2.2%	Repeat angiogram in case of negative initial angiogram
**Nesvick (2021)** (Nesvick et al., 2022)	Retrospective study and meta-analysis	11.1% and 10.4%	0.5% (retrospective part)	Repeat angiogram in case of diffuse bleeding (DSA)
**Bashir (2018)** (Bashir et al., 2018)	Retrospective	1.4%	0.2%	Repeat angiogram in case of nPM-SAH (DSA)
**Little (2007)** (Little et al., 2007)	Retrospective	13%	2.9%	Repeat angiogram (MRI and conventional angiography in case of classic SAH, conventional or non-invasive angiography in case of PM-SAH)
**Mohan (2019)** (Mohan et al., 2019)	Systematic review and meta-analysis	3.9%		No specific recommendation for follow-up imaging
**Nguyen (2022)** (Nguyen et al., 2022)	Retrospective	4.1%	0.9%	Repeat angiogram in case of nPM-SAH (DSA)
**Topcuoglu (2003)**. (Topcuoglu et al., 2003)	Retrospective	4.7%	0.5%	Repeat angiogram in case of nPM-SAH (DSA)
**Ringelstein (2015)** (Ringelstein et al., 2015)	Retrospective	4.5%	0.6%	Repeat angiogram in case of nPM-SAH (DSA)
**Bakker (2014)** (Bakker et al., 2014)	Meta-analysis	10%		Repeat angiogram in case of diffuse bleeding
**Jung KH (2008)** (Jung, 2018)	Retrospective	12.6%	0.6%	Repeat angiogram (DSA in case of nPM-SAH, CTA or MRA in case on PM-SAH)
**Chen (2014)** (Chen, 2014)	Case report and literature review			Further investigations in case of negative angiogram (chirurgical exploration)
**Fontanella (2011)** (Fontanella et al., 2011)	Retrospective	10.8%	1.2%	Repeat angiogram in case of typical bleeding pattern (in case of PM-SAH it is not necessary)
**Xu (2017)** (Xu et al., 2017)	Retrospective	2.9%		Repeat angiogram in case of nPM-SAH
**Wang (2024)** (Wang et al., 2024)	Retrospective	6.4%		Repeat angiogram in intermediate- and high-risk group

*Note*: nPM-SAH: non-perimesencephalic subarachnoid hemorrhage, PM-SAH: perimesencephalic subarachnoid hemorrhage, DSA: digital subtraction angiography.

## 4 Discussion

The rupture of an intracranial aneurysm is considered the leading cause of spontaneous SAH. However, in some cases, no vascular lesion is found on the initial digital subtraction angiography, referred as spontaneous angiogram-negative SAH. This can be attributed to venous hemorrhage or occult aneurysms, which may be masked by vasospasm or massive hemorrhage. While perimesencephalic SAH is often linked to venous bleeding and generally favorable outcomes, undetected aneurysms carry the risk of rebleeding, potentially leading to severe complication (Rinkel et al., 1991). Our review of the literature, encompassing studies from 2000 onward, indicates an initial misdiagnosis rate of 7.8% and an overall incidence of occult aneurysms of 0.8% among all SAH patients. At our institution, we determined a higher incidence rate of 1.1% for angiographically occult aneurysms in aSAH patients, which exceeds the average. This discrepancy may be due to our institutional practice of performing follow-up imaging in nearly all patients with an initially negative angiogram, including those with a perimesencephalic bleeding pattern, particularly in the presence of a severe clinical course.

Few researchers have addressed the question of the modality and timing of repeat angiogram. DSA is considered the gold-standard for cerebrovascular diagnosis, and with technological advancements, the incidence of initial misdiagnosis in aSAH patients has decreased recently (Bakker et al., 2014). Nevertheless, in rare cases, the aneurysm may be not recognized on the initial investigation. For follow-up imaging in such cases, no international accepted guidelines exist. The options for repeat angiography include conventional catheter angiography, CTA or MRA. One study has demonstrated that catheter angiography remains the most sensitive modality for detecting vascular lesions, and in most cases of delayed diagnosis, a vascular lesion was eventually identified on repeat DSA (Topcuoglu et al., 2003). However, the reviewed studies revealed considerable variability in the imaging modalities employed for follow-up, and catheter angiography is still considered the predominant method of investigation. Although CTA and MRA represent non-invasive alternatives with high sensitivity and specificity for aneurysm detection, both modalities have technical and practical limitations. Notably, our cohort included three cases where the aneurysm was identified through MRA, suggesting that non-invasive methods can also be beneficial in specific circumstances, despite DSA remaining the most sensitive tool overall. To our knowledge, no large comparative prospective analysis is currently available evaluating the diagnostic yield of DSA, CTA and MRA specifically in cases of delayed aneurysm detection. Our study highlights the benefit of a repeat-DSA, and in some cases even a third DSA, as 71.4% (n = 10) aneurysms in our cohort were revealed by catheter angiography. Due to the retrospective character of this study and the individualized follow-up approach for each patient, a direct comparison between these imaging tools was not feasible. Although DSA remains the most sensitive tool for aSAH investigation, it is invasive and may carry a risk for complications such as neurological events. Previous reports indicate an overall procedural complication rate of 0-5%, with no complications noted in our cohort (Kumar et al., 2014; Moscovici et al., 2013; Ringelstein et al., 2013; Nesvick et al., 2022; Bashir et al., 2018; Bakker et al., 2014). However, in most cases the detected deficits were transient, and no complications were reported in the remaining articles of our systematic review.

Given the lack of formal guidelines for managing angiogram-negative aSAH cases, decisions regarding follow-up angiography are typically made on a case-by-case basis by the attending physicians or regulated by internal clinic protocols. At our institution, repeat imaging is performed in nearly all patients with an initially negative angiogram. In patients with a perimesencephalic bleeding pattern, this was done particularly in cases with a severe clinical presentation, such as a high HH grade, a large blood volume or hydrocephalus, although the final decision was made on an individual basis. In contrast, multiple studies have suggested that a repeat angiogram may not be necessary in case of perimesencephalic bleeding pattern when there is no evidence of hematoma and vasospasm because of the low incidence among these patients and the benign course of this disease (Kumar et al., 2014; Fontanella et al., 2011). In the four cases with perimesencephalic bleeding patterns and subsequent aneurysm detection at our institute, aneurysms were located at anterior communicating artery in one case, posterior cerebral artery in one case and at basilar artery in two cases. The necessity of further angiographic studies in such cases remains a topic of debate in the literature. Some authors propose that it may be acceptable to follow up patients with perimesencephalic bleeding pattern by noninvasive methods rather than DSA (Little et al., 2007; Jung et al., 2006). In our cohort, four patients with a perimesencephalic bleeding pattern ultimately harbored an aneurysm, three of which were identified by repeat DSA and one by MRA. Although this suggests that a perimesencephalic pattern does not reliably exclude an aneurysmal source, these observations are based on a small number of patients and should be interpreted with caution.

The optimal timing of repeat angiogram is also debatable. Bakker et al. recommended performing a repeat DSA 10 to 14 days after the initial bleeding, as vasospasm may occur more frequently during this period, and thrombosis may obscure the aneurysm (Bakker et al., 2014). Similarly, Fontanella et al. highlighted the increased risk of rebleeding in the early phase post-hemorrhage and advised repeating DSA between the third and tenth day (Fontanella et al., 2011). In our cohort, repeat angiography was performed between one and fourteen days after the hemorrhage (mean: 4.6 days). A third angiogram was performed in three patients at various intervals: two, twelve-, and thirty-days post-hemorrhage. Based on available data, no recommendation can be made regarding the optimal timing for repeat angiography, as it likely depends on patient-specific factors and the occurrence of complications, warranting individualized decision-making.

Our findings show that 21.4% of aneurysms (n = 3) were detected only during a third angiography, indicating that performing additional angiographies can be valuable in specific cases. Among them, one patient presented again to the clinic one month after the initial bleeding with reduced consciousness and memory disturbances. In another patient, recurrent seizures and vasospasm occurred over time, prompting a second repeat angiography. In the third patient, a new-onset neurological deficit led to the performance of repeat DSA. In our opinion, a third angiographic study should not be supposed as a routine follow up examination, but should be rather reserved for selected cases, especially when patients exhibit specific clinical signs, such as poor clinical condition, rebleeding, or pronounced vasospasm, or there are clear radiological patterns suspicious for an occult aneurysm.

Little et al. documented a case in their study in which an aneurysm was discovered through surgical exploration after previous imaging failed to detect any abnormality (Little et al., 2007). A surgical exploration may be warranted in cases where there is a strong clinical and radiological suspicion of an aneurysm. In our cohort, one patient underwent surgical exploration after the second angiogram failed to reveal any vascular lesion; however, radiological findings on CT scan and patient's clinical presentation strongly suggested the presence of an aneurysm. These findings underscore the importance of further investigation in selected cases.

Possible reasons for undetected aneurysms include vasospasm, thrombosis, dissection of the artery, small sized aneurysms or technical errors (Moscovici et al., 2013; Ringelstein et al., 2013, 2015; Nguyen et al., 2022; Khan et al., 2013; Little et al., 2007; Fontanella et al., 2011; Inamasu et al., 2003; Jung et al., 2006; Chen, 2014; Xu et al., 2017). Our findings suggest that rather small aneurysms may be missed on initial DSA. Additionally, high fisher grades were observed in 71.4% of our patients, and atypical aneurysm configurations such as blister-like aneurysms and perforator aneurysms were found in 64.3% of our cases. These findings are consistent with previous studies reporting an increased occurrence of atypical lesions in cases of initially occult aneurysmal subarachnoid hemorrhage (Nguyen et al., 2022). In two of our cases, thrombosis of the aneurysm in one instance and vasospasm in another were identified as possible reasons for the initial inability to detect an aneurysm. In the remaining cases, no specific cause could be determined, though it can be assumed that the very small size of the aneurysms and the presence of a significant amount of blood complicated the initial diagnosis.

We also conducted a detailed analysis of our patient cohort to identify possible predictive factors for angiographically negative aSAH. Patient characteristics were heterogenous within the group in terms of variables such as age, HH score, and medical history. Our findings suggest that previously undetected aneurysms tend to occur more frequently in the anterior communicating artery, as supported by other studies (Nguyen et al., 2022; Inamasu et al., 2003; Jung et al., 2006). In addition, a high fisher grade was commonly observed and may serve as a potential predictive factor for negative initial angiography. Therefore, it could be reasonable to investigate patients with a high amount of blood on the initial CT scan more comprehensively.

An important entity in this context is perforating artery aneurysms, which have increasingly been recognized as a distinct subtype in recent years. These include lenticulostriate artery aneurysms and basilar artery perforator aneurysms. A special classification has been proposed by Satti et al. (2017). These perforating artery aneurysms are often very small and arise from vessels at the limit of angiographic resolution and are therefore frequently missed on initial imaging (Granja et al., 2020; Agarwalla et al., 2014). In this present study, four out of 14 cases were perforating aneurysms, with two BAPAs. Notably, these two BAPAs presented with a perimesencephalic bleeding pattern, indicating that these lesions may be misclassified as non-aneurysmal perimesencephalic hemorrhage.

Regarding diagnostics and evolving techniques, three generations of angiography systems were in use over the study period. Half of our 14 cases occurred after the introduction of the current-generation system, and 3D rotational angiography was performed in most initial examinations. This indicates that very small and atypical aneurysms may escape detection even with state-of-the-art equipment and 3D imaging and that factors such as aneurysm size and morphology, partial thrombosis, vasospasm and surrounding blood may be more relevant than technical limitations alone. At the same time, three of the four perforating artery aneurysms, including both BAPAs, were diagnosed on the current system, which may reflect both improved resolution and growing awareness of this entity.

Complications were noted in 71.4% of patients in our cohort without a significant difference in overall complication rates between the case and control groups. However, three patients (21.4%) in the case group experienced rebleeding before the aneurysm was diagnosed, whereas no rebleeding occurred in the control group. Although the absolute numbers are small and the difference did not reach statistical significance, this finding supports the assumption that patients with an undetected ruptured aneurysm remain at considerable risk of rebleeding (Kumar et al., 2014). As rebleeding is one of the most important factors in determining a poor outcome after aSAH, this underlines the need for timely and thorough repeat diagnostics in patients with an initially negative angiogram.

In conclusion, our study investigated the impact of delayed aneurysm detection in patients with an initially angiographically negative aSAH. Twelve of 14 patients (85.7%) achieved a favorable outcome, and no statistically significant difference in GOS or mRS was found compared with controls with immediate aneurysm detection. However, given the very small sample size, our study was not powered to detect relevant differences in outcome, and the absence of a significant difference should not be interpreted as evidence that delayed detection is without consequence. Importantly, three patients (21.4%) experienced rebleeding before the aneurysm was identified, highlighting the substantial risk associated with an undetected ruptured aneurysm.

### 4.1 Limitations

Nevertheless, several limitations should be acknowledged. First, the small sample size of only 14 patients limits the generalizability of our findings and provides insufficient statistical power to reliably detect differences in clinical outcomes between cases and controls. Second, the retrospective design of the study also poses challenges, as it relies on existing medical data that may be incomplete or misclassified, impacting overall outcomes. In addition, the retrospective design introduces another risk of selection bias: All cases were identified through PACS and our institutional database, so patients with an initially negative DSA who died or were transferred before repeat imaging, could not be included, which may have led to an underestimation of the true incidence.

Third, technical advances in DSA and MRI occurred during the long study period of 17 years. This may as well have affected the detection of occult aneurysms, as not all patients underwent the same follow-up sequence. While this variability may have influenced detection rates, it also reflects the diagnostic workflows of real-world clinical practice. Fourth, although cases and controls were matched for HH grade, Fisher grade, sex and age, residual confounding by unmatched factors such as treatment modality, aneurysm size and location cannot be excluded, particularly given the small sample size.

In conclusion, a large, prospective study is warranted to develop a diagnostic algorithm for cases with an initially negative angiogram.

## 5 Conclusion

In our monocenter cohort of 14 aSAH patients, ruptured aneurysms were detected after a mean of 10 days after an initially negative DSA, while the majority were found on a second or third DSA (71.4%), or MRI in 21.4% of cases. Clinical outcomes were favorable (GOS 4-5) in 85.7% of patients, without a significant difference compared with matched controls. However, given the small sample size and the occurrence of rebleeding in three patients prior to diagnosis, an impact of delayed detection on outcome cannot be excluded.

The detected aneurysms were small (mean size 1.8 mm), with 50% located in the anterior and 50% in the posterior circulation. No associated vascular malformations were noted. Although complications occurred in 71.4% of patients-manifesting as vasospasm, rebleeding, or hydrocephalus-the overall clinical outcome remained favorable for the majority. Nevertheless, undetected aneurysms continue to pose a risk for severe rebleeding and increased mortality. Therefore, repeat angiography should be considered in patients with an initially negative angiogram, including those with a perimesencephalic bleeding pattern, and additional angiographic studies, such as a third catheter angiography, may be beneficial in selected cases.

## Ethics declaration

The authors have NOT obtained informed consent from participants or their legal representatives. It was a retrospective observational study. The data used could not be linked to specific individuals.

This study was performed in compliance with relevant laws, regulatory frameworks and guidelines where the research took place. This study was approved by the Ethikkomission der Technischen Universität München. (Approval No. 186/20S)

## Consent to participate

Informed consent was waived due to the retrospective nature of the study and the use of anonymized clinical data.

## Consent to publish

Not applicable.

## Ethics approval

This retrospective study involving human participants was conducted in accordance with the ethical standards of the institutional and national research committee and with the 1964 Helsinki Declaration and its later amendments or comparable ethical standards. Ethical approval was obtained from the Ethics Committee of the Technical University of Munich (approval number: 186/20S).

## Author contributions

CA, LA and MW contributed to the design of the study. CA and LA were responsible for data curation. Formal analysis was performed by CA and LA. Investigation was performed by CA and LA. Project management was done by MW. Supervision was performed by JK, MW and BM. Validation was performed by AJ, AW, HS, CN, CM, JS, TBB, JK, BM and MW. The original draft was written by CA and LA. All authors contributed to the review and editing.

## Data availability

All source data are securely stored at the Department of Neurosurgery, Technical University of Munich. Researchers interested in data exchange are encouraged to reach out to the corresponding author directly.

## Declaration of generative AI and AI-assisted technologies in the writing process

During the preparation of this paper, the authors utilized ChatGPT4o and ChatGPT5.2 (OpenAI, San Francisco, California, USA) to assist with spelling, grammar, and style corrections. Following the use of these tools, the authors thoroughly reviewed and made additional corrections to ensure accuracy and appropriateness. The authors take full responsibility for the final content and conclusions presented in this publication.

## Funding

No funding for research, writing, and/or publication of this article was received by the authors.

## Conflict of interest

The authors declare that they have no known competing financial interests or personal relationships that could appear to have influenced the work reported in this paper.
